# Clinical Failure After Mitral Transcatheter Edge-to-Edge Repair Mechanisms, Outcomes, and Management

**DOI:** 10.1016/j.shj.2026.101086

**Published:** 2026-07-03

**Authors:** Paul Sorajja, Siddharth Singh, Robert Burke, Philip Gideon, Kenith Fang, David Rizik

**Affiliations:** a Division of Cardiology, Banner University Medical Center – Phoenix Phoenix, Arizona, USA; b Phoenix Cardiovascular Surgery Associates Phoenix, Arizona, USA

**Keywords:** Failure, MitraClip, Mitral regurgitation, M-TEER, PASCAL

## Abstract

Transcatheter repair with edge-to-edge therapy (M-TEER) has been a revolutionary technology for patients with mitral regurgitation (MR). M-TEER is an established safe and effective treatment that alleviates MR, improves quality of life, and, in some patients, improves survival. Nonetheless, a significant number of M-TEER may not have procedural nor clinical success due to a variety of factors, with residual MR and/or mitral stenosis. The clinical manifestation of M-TEER failures may be early or late, with varying degrees of patient impact, ranging from an asymptomatic state to heart failure and poor survival. Because M-TEER patients typically are elderly and with significant morbidity, the management of these clinical failures poses vexing clinical challenges. Herein, we review the incidence and factors that contribute to M-TEER failure, as well as the associated clinical outcomes and management strategies.

## Highlights

• While mitral transcatheter edge-to-edge repair (M-TEER) has been a revolutionary technology, a significant number of patients do not have procedural or clinical success.
• The most common manifestations of M-TEER failure are residual regurgitation or stenosis, and they are often associated with poor survival.
• Traditional surgery is typically high or prohibitive risk due to patients being elderly, having significant morbidity, and meeting the original heart team indications for transcatheter therapy.
• Novel transcatheter therapies for M-TEER failure are emerging, including the ability to fully remove these devices followed by transcatheter mitral valve replacement.
• Herein, we review the incidence and factors that contribute to M-TEER failure, as well as the associated clinical outcomes and management strategies.

## Introduction

For patients with severe, symptomatic mitral regurgitation (MR), mitral transcatheter edge-to-edge repair (M-TEER) has been a revolutionary technology. While many transcatheter mitral repair technologies have failed to achieve clinical success over the past several decades, M-TEER has proven itself as a safe and effective treatment that alleviates MR, improves quality of life, and, in some patients, improves survival. M-TEER is thus the standard of care for patients in need of transcatheter mitral repair as determined by heart team evaluations, with hundreds of thousands of patients already having been treated worldwide.^1^^,^^2^

Nevertheless, there is a significant number of M-TEER patients in whom neither procedural nor clinical success may result. Significant residual or recurrent MR following M-TEER occurs due to a variety of anatomic, procedural, and clinical factors, affecting 10% to 40% of treated patients. In some patients, iatrogenic mitral stenosis may occur with or without MR. Whether due to MR or stenosis, the clinical manifestation of M-TEER failures may be early or late, with varying degrees of patient impact, ranging from an asymptomatic state to heart failure and poor survival. Because M-TEER patients typically are elderly and with significant morbidity, the management of these clinical failures poses vexing clinical challenges.

### Defining M-TEER Failure

While there is no standard definition for M-TEER failure, the Mitral Valve Academic Research Consortium (MVARC) definitions for transcatheter mitral success are highly applicable (Table 1).^3^ Importantly, these standardized definitions are broad in scope and go beyond just the acute procedural result. The MVARC definitions emphasize the relation of transcatheter mitral success to patient recovery, functional improvement, and survival free of procedural complications, heart failure hospitalization, and all-cause mortality. While the MVARC definitions for clinical outcomes are limited to events in the first year after M-TEER, they can easily be extended for long-term analyses. Such an extension is highly important as M-TEER is commonly performed in patients with impaired left ventricular function and with significant morbidity, both of which can contribute to long-term clinical M-TEER failure. Thus, defining the presence of clinical M-TEER failure as the absence of MVARC success is reasonable.

**Table 1:** MVARC definitions of transcatheter mitral success

Technical success (measured at exit from the cardiac catheterization laboratory)	1. Absence of procedural mortality, 2. Successful access, delivery, and retrieval for the device delivery system, 3. Successful deployment and correct positioning of the first intended device, and 4. Freedom from emergency surgery or reintervention related to the device or access procedure
Device success (measured at 30 d)	1. Absence of procedural mortality or stroke, 2. Proper placement and positioning of the device, 3. Freedom from unplanned surgical or interventional procedures related to the device or access procedure; and 4. Continued intended safety and performance of the device, including: a. No evidence of structural or functional failure b. No specific device-related technical failure issues and complications c. Reduction of MR to either optimal or acceptable levels without significant mitral stenosis and with no greater than mild (1+) paravalvular MR (and without associated hemolysis)
Procedural success (measured at 30 d)	1. Device success (either optimal or acceptable), and 2. Absence of major device or procedure-related serious adverse events, including: a. Death b. Stroke c. Life-threatening bleeding (MVARC scale) d. Major vascular complications e. Major cardiac structural complications f. Stage 2 or 3 acute kidney injury (includes new dialysis) g. Myocardial infarction or coronary ischemia requiring PCI or CABG h. Severe hypotension, heart failure, or respiratory failure requiring intravenous pressors or invasive or mechanical heart failure treatments such as ultrafiltration or hemodynamic assist devices, including intra-aortic balloon pumps or left ventricular or biventricular assist devices, or prolonged intubation for ≥48 h i. Any valve-related dysfunction, migration, thrombosis, or other complication requiring surgery or repeat intervention
Patient success (measured at 1 y)	1. Device success (either optimal or acceptable), 2. Patient returned to the preprocedural setting, 3. No rehospitalizations or reinterventions for the underlying condition (e.g., mitral regurgitation, heart failure), 4. Improvement from baseline in symptoms (e.g., NYHA improvement by ≥1 functional class), 5. Improvement from baseline in functional status (e.g., 6-min walk test improvement by ≥50 m), 6. Improvement from baseline in quality of life (e.g., Kansas City Cardiomyopathy Questionnaire improvement by ≥10)

Abbreviation: CABG, coronary artery bypass grafting; MR, mitral regurgitation; MVARC, Mitral Valve Academic Research Consortium; NYHA, New York Heart Association; PCI, percutaneous coronary intervention.

Nevertheless, there are several additional considerations for defining M-TEER failure. These include the need for clinical context and patient individualization, echocardiographic challenges in the assessment of residual disease, and the potential unavailability of other treatment options due to high or prohibitive surgical risk. In this light, qualifying the definition of M-TEER failure with actionable consequences may be helpful.

Herein, we would recommend defining clinical M-TEER failure as any specific device complications (e.g., single leaflet device attachment [SLDA]), residual or recurrent MR, and/or iatrogenic mitral stenosis that drive the decision to seek additional *unanticipated* medical, interventional, or surgical management. For example, a patient with residual MR who had been treated for acute MR and cardiogenic shock as a bridge to cardiac surgery following hemodynamic stabilization would not be a clinical M-TEER failure. Similarly, clinical M-TEER failure would not describe a nonsurgical patient in whom significant clinical improvement occurs despite residual MR. Conversely, clinical M-TEER failure would be present in a patient originally treated successfully but in whom symptomatic, severe secondary MR arises from subsequent myocardial infarction and left ventricular dysfunction. Because adjuvant therapy for heart failure is important for clinical success with M-TEER therapy, the occurrence of adverse events, such as heart failure hospitalization or death, that happen despite durable reduction in MR is also reasonable. Context that underlies the reasons for the need to manage unsatisfactory M-TEER results is elementary to both the understanding of the causes and determining the clinical importance of the failure when it occurs.

### Assessment for Clinical M-TEER Failure

For patients presenting with possible M-TEER failure, the symptoms should be attributable to significant mitral pathology. Symptoms may reflect left-sided heart failure, such as dyspnea, but could also be from low cardiac output and manifest only as fatigue. As both a mechanism and consequence of its therapeutic effect, M-TEER device placement typically reduces the anteroposterior diameter of the mitral valve with a corresponding reduction in the overall mitral valve area. Residual or recurrent MR as well as mitral stenosis can occur after M-TEER, with the symptomatology usually related to whether regurgitation or stenosis predominates or both if there is mixed disease.

Echocardiographic assessment of residual or recurrent MR is fundamental for the assessment of potential M-TEER failure, but it can be challenging. Color-flow Doppler is the most common method due to its simplicity, with the absence or minimal presence of color flow highly indicative of mild or no residual MR.^4^ Limitations of color flow Doppler arise when there is splaying of the jet despite significant MR reduction (i.e., the “garden hose effect”) or when the jets are multiple, in different planes, or merged. A wide vena contracta or large proximal flow convergence on the left ventricular side helps indicate the presence of more than mild residual MR.

Measurement of effective regurgitant orifice area by the proximal isovelocity surface area method is not recommended following M-TEER due to the need for angle correction and frequent multiple jets. As a better alternative method, 3-dimensional (3D) imaging of the vena contracta area (VCA) is commonly performed and has a strong correlation with quantitation performed by cardiac magnetic resonance imaging. The VCA for each jet is analyzed separately and added for quantitation. VCA values greater than 0.20 cm^2^ suggest that more than mild MR is present. All comprehensive echocardiography evaluations should also include assessment of pulmonary vein flow patterns (e.g., change in S-wave reversal or blunting to antegrade flow), 3D color imaging (especially for eccentric jets), and improvement in forward stroke volume. Strain assessment for left atrial and left ventricular function has also been shown to carry prognostic information.

Placement of 1 or more M-TEER devices changes the mitral valve orifice from singular to multiple with varying sizes. Due to the different sizes of the mitral orifices following M-TEER, hemodynamic assessment for the mitral gradient is performed using mean values (not peak). Due to the flow-dependent nature of diastolic mitral gradients, notation of the cardiac output or heart rate should be made. In selected patients with concerning symptoms and without evidence of severe gradients at rest, a study to assess for hemodynamically significant mitral stenosis with exercise should be considered. Planimetry of the mitral valve area using 2-dimensional (2D) and 3D methods also provides reliable, complimentary information regarding the presence of significant stenosis.

### Causes of M-TEER Failure

While technical failure may occur from any part of the procedure, the focus of this review is on device-specific causes (Figure 1). These causes can be grouped broadly into anatomic, procedural, and patient factors. However, critical to the achievement of clinical success and avoiding M-TEER failure is an understanding of the intended clinical effect of the therapy.

**Figure 1:**
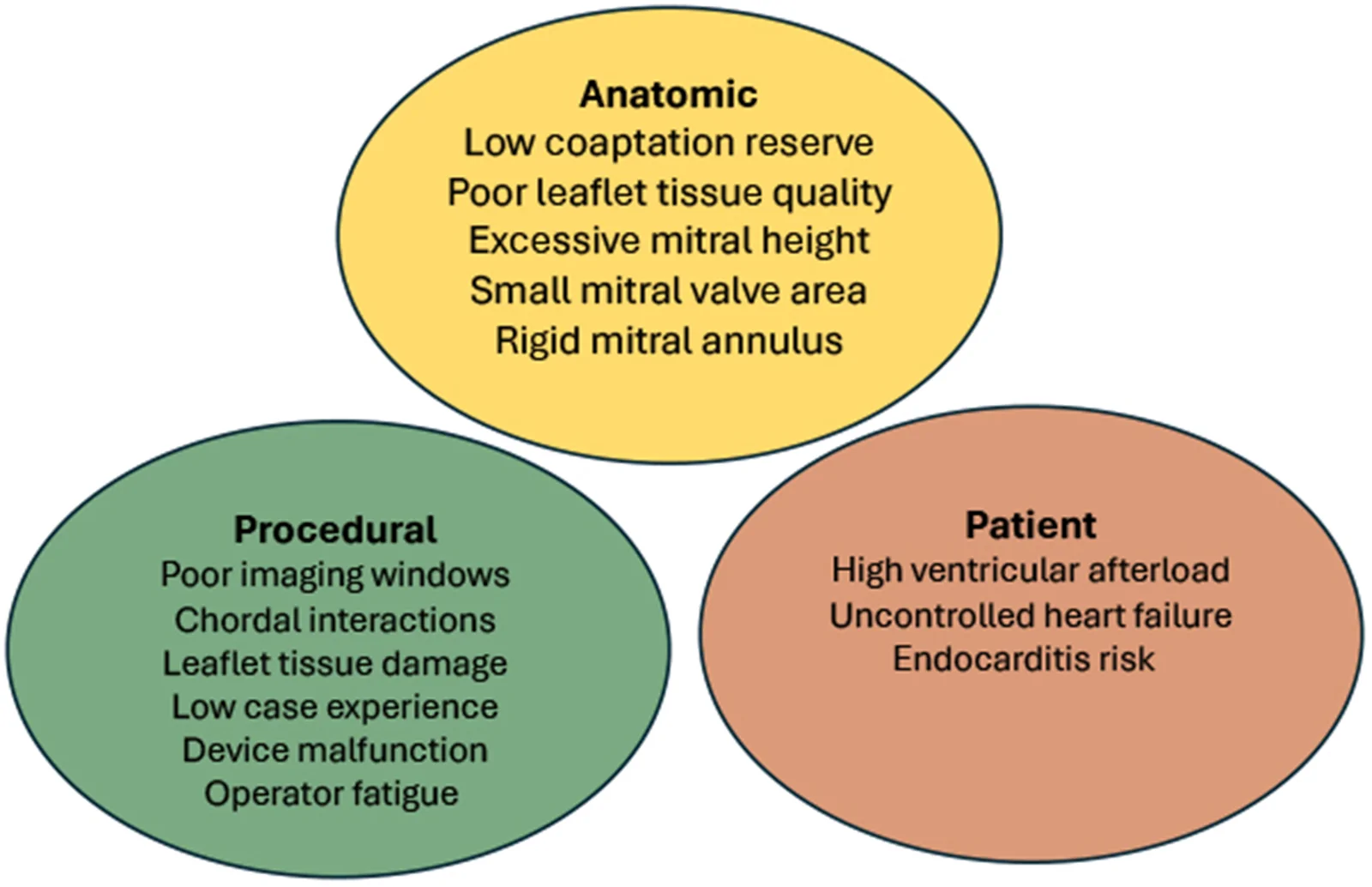
**Contributory factors for failure with mitral TEER therapy.** Abbreviation: TEER, transcatheter edge-to-edge repair.

In normal mitral valves, a natural state exists where >7 mm of leaflet coaptation is available to prevent regurgitation (Figure 2). Like a keystone in a Roman arch, this coaptation reserve is effective under varying ventricular loading conditions, with the functional aspects of the coaptation plane being anterior-posterior for the mitral valve. Thus, a key objective for treating and preventing MR, by any therapy, is augmenting coaptation reserve. The augmentation in coaptation reserve may come from reduction in valvular height, leaflet volume, or technical apposition, all of which are considered relatively segmental in their approach. Methods that reduce annular area can improve the entire coaptation plane and enhance the durability of the repair result. Mitral leaflets with insufficient augmentation in coaptation reserve simply will have residual regurgitation and/or be at high risk of recurrent disease. MR, whether primary or secondary, arises from etiologies that are dynamic and progressive. Thus, unaddressed pathology in terms of leaflet volume and height, as well as uneven areas of valvular tension, also predisposes to the risk of recurrent disease.

**Figure 2:**
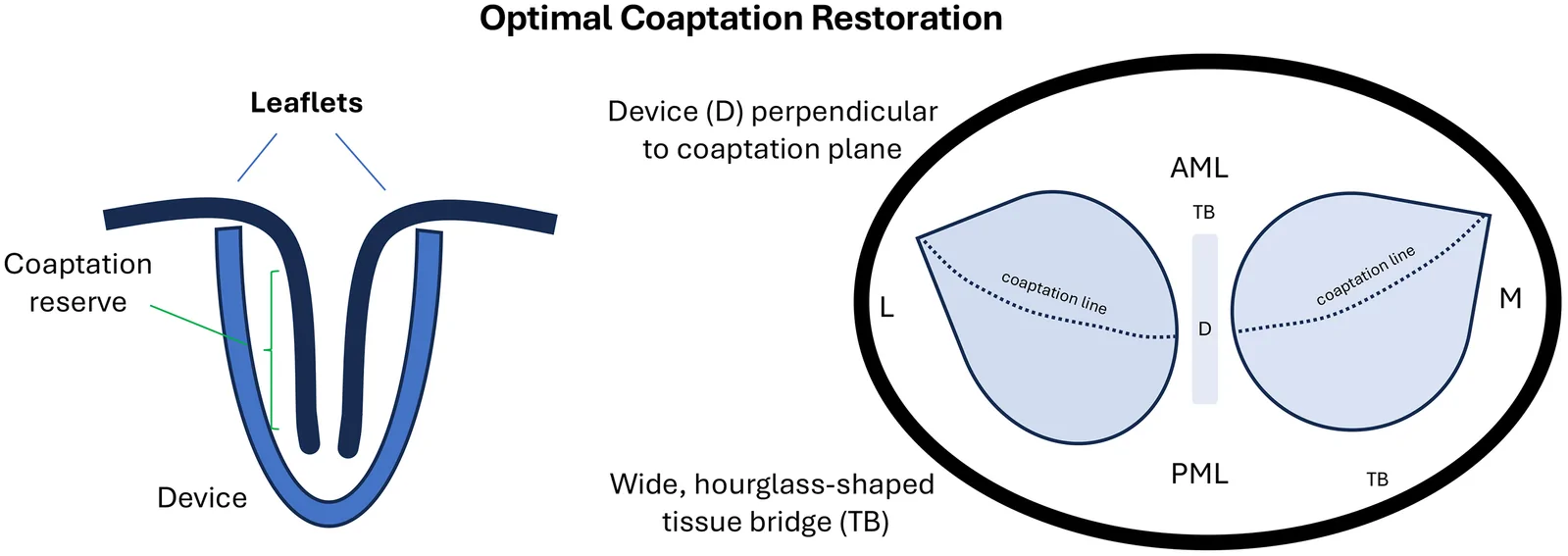
**Transcatheter restoration of coaptation reserve.** Abbreviations: AML, anterior mitral leaflet; D, device; L, lateral; M, medial; PML, posterior mitral leaflet; TB, tissue bridge.

### The Misleading Term of “TEER”

The most commonly used M-TEER technologies in current practice are MitraClip (Abbott Laboratories, Chicago, IL) and PASCAL (Edwards Lifesciences, Irvine, CA). While the results of these therapies have remarkably evolved to a high degree of effectiveness and safety, there is still room for a broader improvement in clinical outcomes.

A fundamental issue is that these devices have been described as transcatheter edge-to-edge repair (TEER) for treating both mitral and tricuspid regurgitation. The term “TEER,” in our opinion, is an overly reductive description that mischaracterizes the therapeutic aim of the technology. In various settings, these therapies have been described as a “percutaneous Alfieri stitch,” but such mischaracterization can be harmful. For example, when operators focus only on tying leaflets together, suboptimal results can arise from inadequate leaflet insertion, curling, and pinwheeling, as well as from ignoring the effects (or lack thereof) of device placement on adjacent valvular pathology and coaptation planes (Figure 3). In these situations, some degree of reduction in regurgitation might occur (i.e., an “acceptable” result) but hardly be the ultimate desired goal.

**Figure 3:**
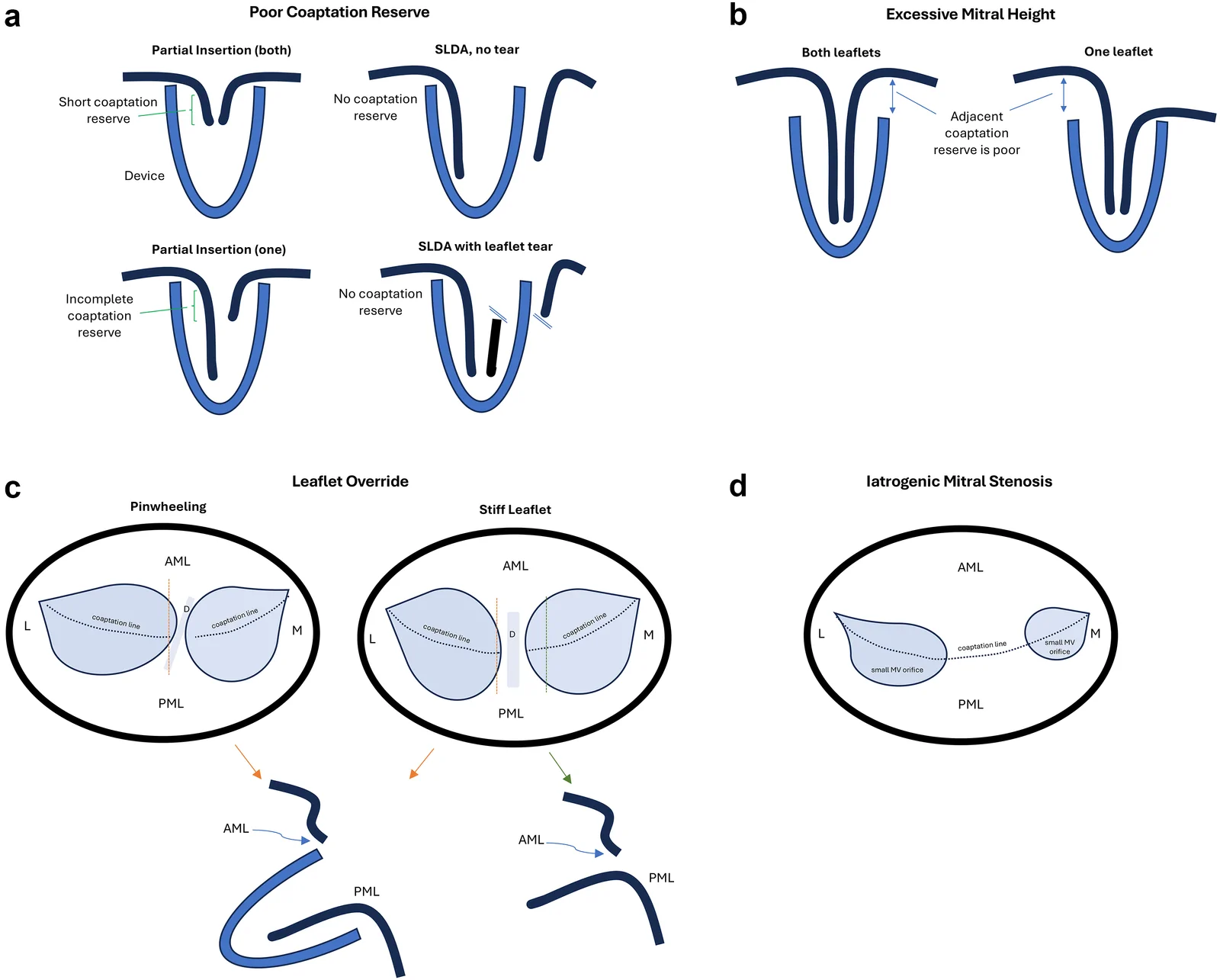
**Common causes of mitral TEER failure.** For each of these categories, anatomical and procedural factors contribute to the failure. **(a)** Poor coaptation reserve results from poor baseline reserve (e.g., severe atrial functional mitral regurgitation), poor leaflet insertion, and SLDA, which can occur with or without leaflet tear or injury. **(b)** Excessive mitral height can occur from poor insertion but also commonly occurs severely degenerative disease. Degenerative disease can be present at baseline or occur in clinical follow-up. **(c)** Leaflet override occurs when 1 leaflet is relatively fixed in relation to the opposing leaflet. This situation can occur with pinwheeling or leaflets with poor compliance (i.e., stiff leaflets). **(d)** Iatrogenic mitral stenosis can be present immediately after the device is implanted or from excessive tissue overgrowth in clinical follow-up Abbreviations: AML, anterior mitral leaflet; D, device; L, lateral; M, medial; PML, posterior mitral leaflet; SLDA, single leaflet device attachment; TB, tissue bridge; TEER, transcatheter edge-to-edge repair.

By defining these therapies as TEER, the operators therefore can lose sight of long-standing principles developed from cardiac surgery for the most effective means of treating atrioventricular regurgitation. Getting surgical-like results requires a focus on transcatheter coaptation restoration, also known as TCR, an acronym that perhaps better reflects what we are trying to achieve clinically. Importantly, these principles can be applied to any transcatheter therapy for the purposes of treating mitral (or tricuspid) regurgitation.

### Anatomic and Procedural Considerations

Focusing on the restoration of coaptation and augmenting its reserve benefits the operator for both patient selection and procedure outcomes. Essentially, better clinical results occur when there is better coaptation at baseline and when significantly augmenting said coaptation is technically possible. For example, while ∼2 mm of baseline coaptation has been used as a consideration for MitraClip eligibility, a threshold of ∼3.5 mm more recently has been found to be predictive of mild or no residual regurgitation.^5^ Common categories for clinical failure are described in Figure 3.

For assessing the potential to augment coaptation, considerations are given to the leaflets in terms of their symmetry, height (e.g., total and relative annular distance), lengths (e.g., amount for device insertion), orientation (e.g., posterior), mobility (e.g., flat, tethered), and pathology (e.g., calcification, friability), as well as to the mitral annulus in terms of its size and flexibility (i.e., calcification). Flexibility of the mitral annulus is important because reductions in the cross-sectional diameter will improve leaflet coaptation across the entire plane, with a rate of improvement of ∼1 mm for each ∼2 mm reduction in that diameter. Annular flexibility is an important consideration even for leaflet apposition therapies such as MitraClip and PASCAL, as these have been associated with annular reduction in selected patients.

Device selection, now available in multiple sizes from multiple vendors, enables tailored matching of the therapy to the leaflet pathology and treatment goals. Overall, in a recent analysis with MitraClip, ∼7 mm of augmentation in coaptation was present in those with optimal reduction in MR (i.e., mild or no residual), but patients with moderate residual regurgitation only had ∼3.0 mm. Moreover, among patients undergoing MitraClip, greater reductions in mitral annular area, anterior-posterior diameter, or commissural diameter are known to be associated with a greater likelihood of an optimal reduction in MR and associated survival.

Imaging to guide appropriate leaflet insertion is elementary in these procedures. Multiplanar reconstruction and 3D imaging have greatly enhanced procedure performance. In our practice, multiplanar reconstruction is used for gross steering maneuvers. However, for leaflet insertion, we prefer the high-resolution capability of 2D imaging as an important check prior to device release. Using the highest resolution possible helps to ensure deep and taut leaflet insertion into the device, avoid partial leaflet insertion (i.e., side biting), and assist in distinguish chords from leaflets. Also, an often overlooked indicator of poor leaflet insertion is the presence of color flow through the device despite its complete closure.

Patients in whom significant and sustainable augmentation in coaptation reserve is not achievable are at risk of residual MR after M-TEER and having clinical failure. Multiple studies have shown a relation between the acute residual MR and a higher probability of progressive or recurrent MR, as well as the need for repeat intervention in follow-up.^6^ Patient selection and procedural performance to restore the mitral valve to its state of natural coaptation as much as possible without iatrogenic stenosis helps with acute and durable therapy success.

### Patient Factors

National practice guidelines recommend that M-TEER be performed on a background of optimal medical therapy (OMT). OMT is particularly relevant for patients with impaired left ventricular systolic function and in whom progressive ventricular dilatation carries a heightened risk of recurrent MR. When the use of OMT is rigorous, the risk of recurrent MR is low. For example, in the Cardiovascular Outcomes Assessment of the MitraClip Percutaneous Therapy (COAPT) trial, where OMT was mandatory, durable MR reduction was observed (i.e., ∼94% with MR ≤ 2) and repeat mitral interventions were low (e.g., 3.9% over 4-year follow-up).^7^ Conversely, different findings are evident in real-world registries of patients with secondary MR treated with M-TEER. These latter registries have shown survivors at risk of progressive ventricular dilatation and recurrent MR, particularly if not on adequate OMT at baseline.^8^^,^^9^ Thus, various means of mitigating ventricular remodeling, including medical and device therapy, would be expected to minimize the risk of recurrent or progressive MR and clinical M-TEER failure.

### Clinical Impact of M-TEER Failure

Residual or recurrent MR following M-TEER has long been associated with poor clinical outcomes (Table 2).^10^, ^11^, ^12^, ^13^, ^14^, ^15^, ^16^, ^17^, ^18^, ^19^, ^20^ Overall, the 1-year mortality rate for severe MR occurring despite M-TEER is very high, ranging from 30% to 50%. For heart failure hospitalization, the incidence at 1 year is 25% to 50% in afflicted patients. The association with poor outcomes is strongest for severe MR, but several studies have also shown that even moderate disease may be predictive.

**Table 2:** Selected studies on the clinical impact of M-TEER failures

Author	Study description	Key findings	Other
Stocker et al. (2024)^8^^,^^19^	EuroSMR registry of SMR patients (n = 1628)	1-y survival for MR grade ≤1, grade 2, and grade ≥ 3 was 76.4%, 68.7%, and 62.7%, respectively. Residual MR was also predictive of 5-y survival.	Predictors of 5-y mortality were age, renal function, residual MR, NYHA functional class, LVEF, and COAPT eligibility.
Makkar et al. (2023)^20^	STS/TVT registry of DMR patients (n = 19,088)	Lowest mortality was in patients with both ≤mild MR and mitral gradient ≤5 mmHg.	Acute M-TEER failure was associated with 1-y mortality of 26.7%.
Sorajja et al. (2017)^11^	STS/TVT registry of all M-TEER patients (n = 2952)	One-y death rate for grade 3 or 4 residual MR was 48.9%.	
Benfari et al. (2022)^17^	MitraSwiss registry of DMR patients (n = 1187)	1-y mortality with residual grade 3 or 4 vs. trivial MR was 30% vs. 11% (*p* < 0.0001).	Acute procedural failure occurred in 7.6% overall.
Ludwig et al. (2025)	PRIME XL registry (n = 3083)	2-y death or HFH was 29.1%, 41.7%, and 58.0% for MR ≤ 1+, 2+, and ≥ 3+, respectively.	MG > 5 mmHg was not linked to the outcome.
Mustafa et al. (2025)^18^	REPAIR registry (n = 2172)	One-y mortality was highest (20.1%) when both residual MR grade ≥2 and MG ≥ 5 mmHg.	One-y mortality was significantly lower (8.7%) when both mild residual MR and MG < 5 mmHg was present.
Neuss et al. (2017)^10^	Single-center report (n = 268)	MG > 5 mmHg related to combined endpoint that includes death and MV reintervention.	MG > 5 mmHg was present in 25% of the entire cohort.
Patzelt et al. (2019)^15^	Single-center report (n = 255)	Residual MR was predictive of the combined endpoint. MG > 4.4 mmHg was also predictive in DMR patients but not FMR patients.	
Koell et al. (2022)^12^	Single-center report (n = 713)	MG > 5 mmHg related to 5-y death or HFH in DMR patients but not FMR patients.	MG > 5 mmHg was present in 37% of the DMR patients.
Yoon et al. (2022)^14^	Single-center report (n = 419)	Quartiles of MG was not related to 2-y outcomes.	There were very few patients with severe residual disease after M-TEER (3%).
Halaby et al. (2021)^13^	COAPT trial analysis (n = 250)	Quartiles of MG were not related to 2-y outcomes.	There was a high degree of MR reduction that was similar and durable for all 4 mitral gradient quartiles.

Abbreviations: COAPT, Cardiovascular Outcomes Assessment of MitraClip Percutaneous Therapy; DMR, degenerative mitral regurgitation; FMR, functional mitral regurgitation; HFH, heart failure hospitalization; LVEF, left ventricular ejection fraction; MG, mean mitral gradient; MR, mitral regurgitation; M-TEER, mitral transcatheter edge-to-edge repair; MV, mitral valve; NYHA, New York Heart Association; SMR, secondary mitral regurgitation; STS, Society of Thoracic Surgeons; TVT, transcatheter valve therapy.

For iatrogenic mitral stenosis, several early analyses reported poor outcomes with mitral gradients >4 mmHg after M-TEER. However, the data on the significance of these mitral gradient thresholds have been conflicted, especially when comparing the impact of such thresholds vs. that attributable to residual MR. For example, in the large PRIME XL registry, where 3083 patients treated with M-TEER were analyzed, death or heart failure hospitalization at 2-year follow-up was linked to worse residual MR (29.1 vs. 41.7% vs. 58.0% for MR ≤ 1+, 2+, and ≥3+, respectively), but not to the presence of mitral gradients of >5 mmHg.^20^ Residual MR severity, but not a mitral gradient >5 mmHg, also was related to functional improvement as measured by New York Heart Association functional class. Notably, in the studies that have analyzed the clinical impact of mitral gradients after M-TEER, very few of these patients had gradients in the severe range (i.e., >10 mmHg), and stress testing to examine for functional mitral stenosis was not reported.

Importantly, the relation of residual mitral pathology after M-TEER to poor outcomes is often described in strong, causative terms, but significant confounders should be noted. While operator performance certainly contributes to procedural failure, residual mitral pathology also is strongly related to worse baseline disease and a poor patient protoplasm that contribute to impaired survival. Because these patients often have no other therapeutic option due to their high or prohibitive surgical risk, many M-TEER procedures are being performed on mitral pathology that perhaps would be better treated with replacement rather than an attempt at repair. Of note, several analyses have described that the vast majority of patients who have M-TEER (i.e., 60% to 70%) do not have “green-zone” criteria. Whether associate or causative, the impact of residual or recurrent MR after M-TEER is nonetheless highly significant, with symptomatic patients in need for therapeutic options.

### Management

For patients with clinical M-TEER failure, repeat intervention may be considered for those with refractory symptoms and significant mitral disease. In these scenarios, key therapeutic considerations have been a repeat M-TEER procedure, open cardiac surgery, or alternative percutaneous electrosurgical approaches.

### Repeat M-TEER

Overall, repeat M-TEER procedures occur in 4% to 10% of all patients, primarily for those with addressable residual or recurrent MR.^10^, ^11^, ^12^, ^13^, ^14^, ^15^, ^16^, ^17^, ^18^, ^19^, ^20^, ^21^, ^22^ Notably, while success is achievable in the majority of repeat M-TEER procedure cases (i.e., 60% to 80%), these patients are few and highly selected, and those with significant stenosis are not eligible for this approach. Not unexpectedly, success rates with repeat M-TEER are significantly lower than that observed with initial procedures. This lower success rate with repeat procedures is related to the complexity of the mitral pathology that contributed to the M-TEER failure (e.g., poor leaflet integrity, worse flail height, etc.) as well as limitations on placement due the presence of pre-existing devices (e.g., presence of SLDA, higher mitral gradient, or small mitral valve area, etc.). The anatomical selection for repeat M-TEER therapy is otherwise similar to initial procedures, with the primary goal being restoration of coaptation reserve to relieve MR without causing significant iatrogenic stenosis. We have found that repeat M-TEER therapy is most successful for patients with sufficient leaflet quality, physical space for adjacent device therapy, and a permissible mitral valve area. Distinguishing mitral gradients due to large flow related to severe MR from mitral gradients due to small mitral valve area in these considerations is important, as the former but not the latter may be a treatable scenario.

### Cardiac Surgery

Cardiac surgery is one of the most common approaches used to treat patients with clinical M-TEER failure but still remains relatively infrequent. The most comprehensive report on cardiac surgery after M-TEER is from the Society of Thoracic Surgeons database, which examined 463 patients (median age, 76 years) treated between 2014 and 2020.^23^ Operative mortality was 10.2%, higher than the Society of Thoracic Surgeons–predicted mortality of 6.5%, and was significantly related to case volume (i.e., 2.6% vs. 12.4% for >10 cases vs. ≤10 cases, respectively). Of note, the surgical repair rate was 4.8% overall and 6.8% in the subset of patients with degenerative disease.

The operative mortality is high in these patients, as the majority are already at significant surgical risk before M-TEER, and this risk only increases when M-TEER failure occurs and manifests as symptomatic residual mitral pathology. While repair is generally preferred over replacement in surgical approaches to MR, this preference is not evident for M-TEER patients for a variety of reasons. First, these patients are often elderly and at high or prohibitive surgical risk. Thus, the survival benefit of repair over replacement is not as evident in this cohort compared to younger, healthier patients. Second, in elderly patients or those with significant morbidity, reducing on-pump time is preferred, and this is easily achievable with a planned mitral valve replacement. The chronicity of the implant may lead to more tissue overgrowth, subvalvular scarring, changes in mitral pathology, and thus greater challenges in removing these devices. Third, many M-TEER patients have secondary MR, which is known to be associated with a risk of recurrent MR after surgical repair is performed.^24^^,^^25^ Patients who have M-TEER failure are difficult to repair by most surgeons, and a poor repair has a much worse clinical impact than a sufficient replacement. Moreover, surgical repair after M-TEER has been described in selected cases. The ability to surgically repair patients after M-TEER failure is related to the amount of undamaged mitral leaflets. Of note, for the removal of MitraClip devices, pulling on the lock harness with a threaded suture can unlock the device and help remove it with minimal collateral damage.

### Percutaneous Occluder Placement

For patients with residual or recurrent MR after M-TEER, percutaneous placement of occluder devices can be considered. Overall, the technique works best when there are multiple TEER devices in close approximation. Placement is sometimes performed in the mitral commissures, lateral or medial to the TEER devices, but the results can often be suboptimal due to the eccentric nature of this space. For selected cases, a variety of such devices have been utilized, including the Amplatzer Vascular Plug (AVP), Ductal Occluder, CardioForm, and Amplatzer Septal Occluder. Placement of these occluder devices is typically performed using a steerable catheter in the left atrium (e.g., 8.5-French [Fr] Agilis), followed by wiring of the target area with a coronary guidewire or Glidewire. Device delivery can be performed with coronary guide catheters (e.g., 6–8 Fr multipurpose or JR) or Flexor sheaths (Figure 4). We typically recommend straddling of the device in both the left atrial and left ventricular aspects of the location of the MR jet. For devices with more than 2 segments (e.g., AVP II), repositioning of the 2 segments to the alternate chamber may be required to achieve the desired effect (i.e., 2 lobes in the left atrial side vs. 2 lobes in the left ventricular side).

**Figure 4:**
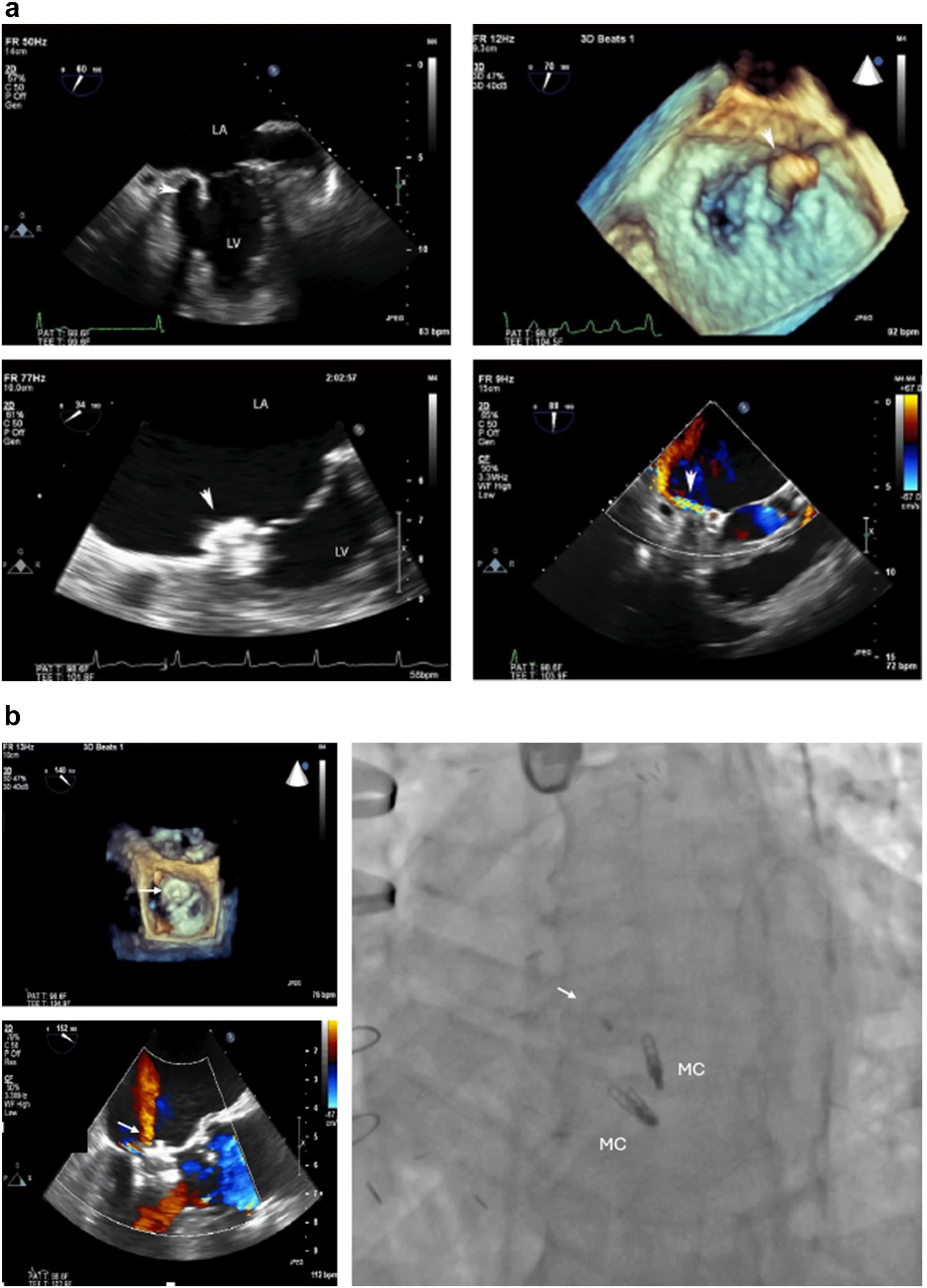
**Case examples of occluders for M-TEER failure. (a)** This patient had recurrent MR after placement of a MC with residual MR located medial to the device (top left, arrow). A steerable guide catheter (top right, arrow) and Glidewire were used to cross medially, followed by insertion of a 6-Fr multipurpose guide catheter. A 12-mm AVP II was placed (bottom left, arrow) with mild residual MR (bottom right, arrow). **(b)** This case shows a 12-mm AVP II occluder that was placed between 2 prior MitraClip devices (top left, arrow) with mild residual MR (bottom left, arrow). The occlude is visible on fluoroscopy between the 2 devices (right, arrow). Abbreviations: AVP II, Amplatzer Vascular Plug II; Fr, French; LA, left atrium; LV, left ventricle; MC, MitraClip; MR, mitral regurgitation.

### ELASTA-Clip

For transcatheter treatment of M-TEER failure, electrosurgical laceration and stabilization (ELASTA-Clip) was originally described in 2019.^26^ In this approach, 2 steerable guide catheters are used to position a partially denuded coronary wire (i.e., 0.014″ Astato) with a flying “v” at the tissue bridge of the previously implanted M-TEER devices, followed by electrosurgical laceration of the bridge and subsequent transcatheter mitral valve replacement (TMVR, Figure 5). ELASTA-Clip, in effect, creates one or more SLDAs, which are then jailed by the TMVR prosthesis. For transapical approaches, the flying “v” also can be created in the left atrium from a DrySeal sheath in the left ventricular apex.^27^ In some cases, an Ono basket has been used to help with M-TEER device capture and full retrieval.^28^

**Figure 5:**
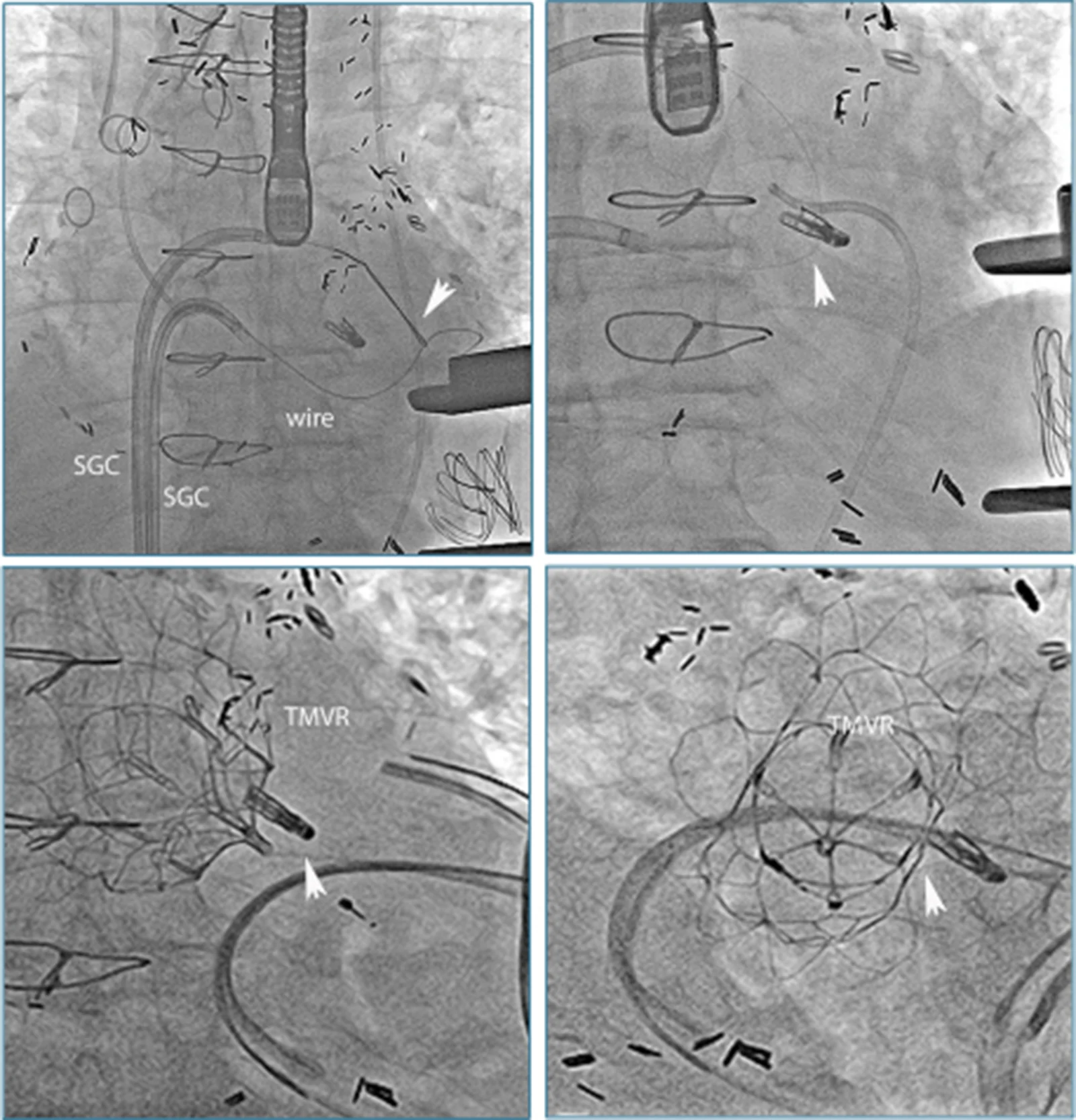
**The ELASTA-Clip procedure.** Top left: Using 2 SGCs, a wire is looped around the tissue bridge and snared (arrowhead). Top right: The flying V is apposed against the tissue bridge followed by electrosurgery to lacerate the bridge. Bottom left and bottom right: the device (arrowheads) is jailed as single leaflet device attachment by the TMVR prosthesis. Abbreviations: SGC, steerable guide catheter; TMVR, transcatheter mitral valve replacement.

The procedure has been successfully performed with a variety of transapical and transseptal TMVR devices (e.g., Tendyne, Intrepid, M3, AltaValve). In a multicenter experience with Tendyne TMVR (n = 22), technical success rates for ELASTA-Clip were reported to be 95% without left ventricular outflow tract obstruction or need for surgery.^29^ ELASTA-Clip has the advantages of being a transcatheter approach but has limitations for anatomic eligibility (i.e., laceration of anterior bridges only), ventricular arrhythmias (i.e., due to stimulation by the residual SLDAs), and issues attributable to off-label techniques, such as procedural complexity and risk of stroke. In some patients, paravalvular regurgitation can occur from collateral damage to the mitral apparatus (i.e., inadequate sealing) or from physical displacement of the TMVR prosthesis by the M-TEER devices left behind.

### AMX Technologies

The AMX TEER management system (AMX Technologies, Maple Grove, MN) is a technology that is specifically designed for electrosurgical removal of failed mitral and tricuspid TEER devices (Figure 6). The system consists of a 24-Fr steerable guide, electrosurgical catheters in varying shapes (right-handed and left-handed) and configurations (i.e., single and multiple cutting electrodes), and a retrieval basket for protection during TEER device removal. As a purpose-built technology, the AMX system offers the ability to fully remove implanted TEER devices with minimal collateral damage. The AMX system has been used successfully in 7 patients thus far on a compassionate use basis (i.e., 6 mitral cases and 1 tricuspid case), with plans to begin a formal early feasibility study in 2026.

**Figure 6:**
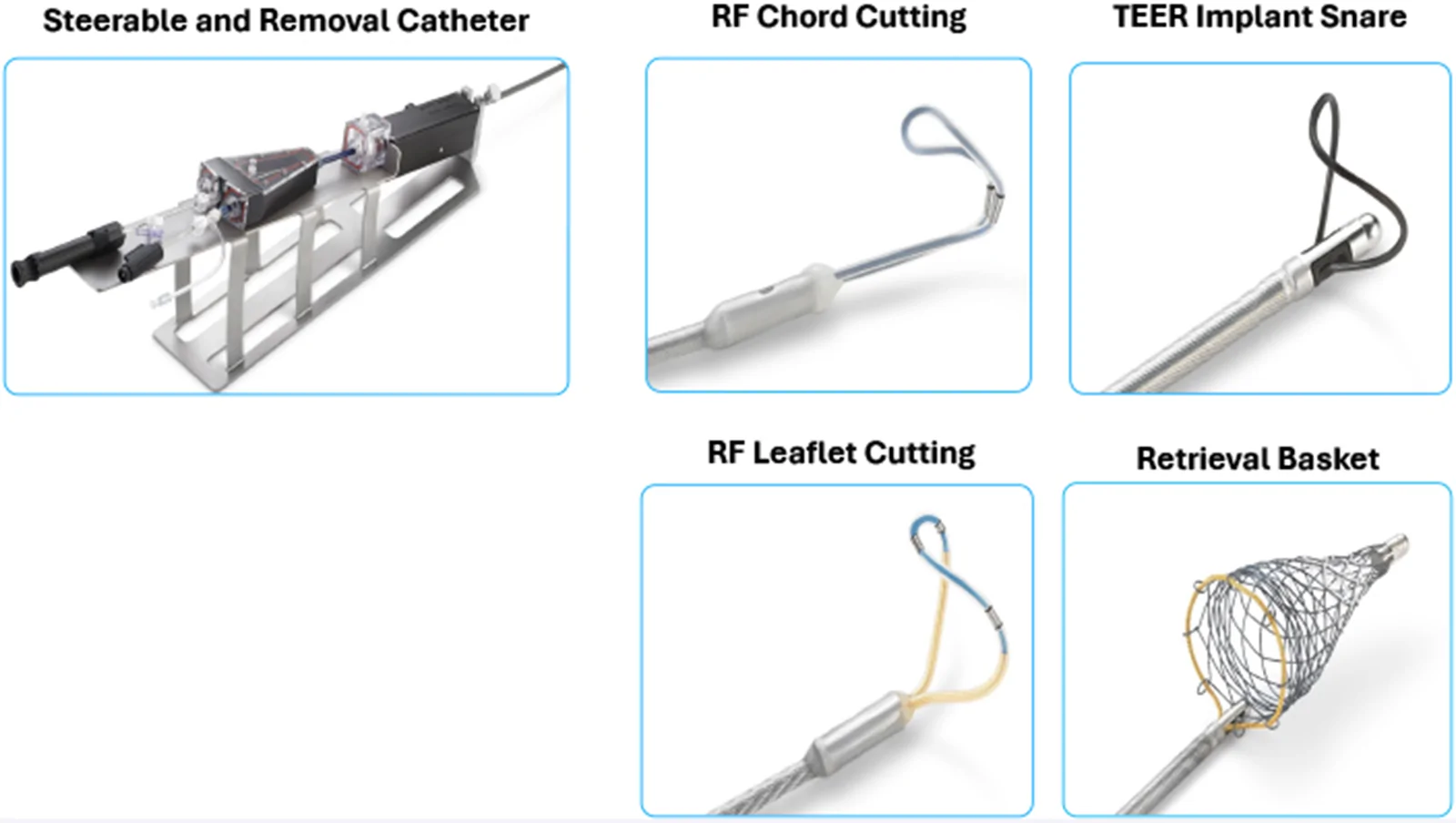
**AMX Technologies LCMS.** The system is a device specially designed for electrosurgery and removal of TEER devices. The 24-Fr system consists of a steerable catheter, snare, retrieval basket, and dedicated cutters for chords and leaflets. The device remains under investigational use. Abbreviations: Fr, French; LCMS, leaflet cutting management solution; RF, radiofrequency; TEER, transcatheter edge-to-edge repair.

## Conclusions

Patients who have clinical M-TEER failure are common and have a poor prognosis if left untreated with a 1-year mortality of 30% to 50%. These patients present with significant residual or recurrent MR or with iatrogenic mitral stenosis, leading to complex clinical challenges. Management of these patients is challenging due to poor mitral pathology that limits the ability to perform repeat M-TEER, poor surgical risk that leads to a high operative mortality, and transcatheter options that are largely limited to complex, off-label techniques. Future technological development to address the unmet needs of this space remains ongoing.

## Funding

The authors have no funding to report.

## Disclosure Statement

R. Burke reports consulting relationship with Siemens Healthineers. K. Fang reports speaker fees from Abbott Structural and Medtronic. P. Sorajja reports fees for consulting from 4C Medical, Abbott Structural, Adona, Arcos, Boston Scientific, ConKay Medical, Coramaze, CroiValve, Cultiv8, Edwards Lifesciences, GE Medical, Haemonetics, InQ8, Medtronic, Philips, Polares, Tricares, VDyne, Unorthodox Ventures, Valcare, and xDot. The other authors had no conflicts to declare.
